# The Most Common Multi-Drug Resistant Bacteria Associated with Hospital Infections, in Urmia, Iran

**DOI:** 10.30699/ijp.2024.2014294.3195

**Published:** 2024-07-24

**Authors:** Seyyed Jalil Mousavi, Rahim Nezhadrahim, Farima Abdulzadeh

**Affiliations:** 1 Department of Infectious Diseases, Urmia University of Medical Sciences, Urmia, Iran

**Keywords:** Drug Resistance, Escherichia coli, Nosocomial Infections, Prevalence

## Abstract

**Background & Objective:**

Hospital-acquired infections (HAIs) are a major healthcare problem in hospitalized patients, especially in developing countries, where they affect millions of patients and cause high mortality rates. This study aimed to investigate multidrug-resistant bacterial strains in NIs at Imam Khomeini University Hospital in Urmia, Iran.

**Methods:**

This cross-sectional study was conducted using a convenience sampling method. The study population comprised all positive clinical samples from HAIs registered in the laboratory of Imam Khomeini Hospital, Urmia, Iran, in 2019. Bacteria were identified by culturing the samples on blood agar and MacConkey agar, followed by performing standard biochemical tests. Antibiotic susceptibility testing was carried out using the disk diffusion method, in accordance with CLSI guidelines.

**Results:**

Of the 607 positive samples, the most common microorganisms isolated were *Escherichia coli* (27.5%), *Acinetobacter baumannii* (18.5%), and *Klebsiella pneumoniae* (15.2%). The distribution of resistance to the number of antibiotics in bacterial isolates from the samples showed that 19.8% of them were resistant to one antibiotic and 13.2% were resistant to three antibiotics. 40.5% of the samples showed no resistance to antibiotics.

**Conclusion:**

This study highlights the critical issue of HAIs and the prevalence of multidrug-resistant bacteria in Urmia, Iran. Urgent measures, including improved hygiene, accurate diagnostics, appropriate antibiotic use, and stakeholder education, are essential. Establishing a robust HAI surveillance system is also recommended. Future efforts should aim at understanding and mitigating the spread of these pathogens.

## Introduction

Nosocomial infections (NIs) are a major challenge for healthcare systems worldwide. According to the World Health Organization (WHO), approximately 9% of hospitalized patients in developing countries develop infections acquired during their stay in the hospital (1). These infections also cause more than 700,000 deaths annually (2). In addition to increased mortality, NIs lead to longer hospital stays, increased healthcare costs, and strain on healthcare systems. Studies have shown that NIs can increase healthcare costs by up to fourfold (3). In the United States alone, NIs result in 75,000 deaths and over USD 9.8 billion in additional healthcare costs each year (4). This contrasts with the relatively low cost of infection control measures. NIs also increases the risk of transmission to other patients and can lead to increased morbidity and mortality. NIs have been shown to triple the mortality rate in hospitals (5). Some of the common types of NIs are surgical site infections (SSIs), ventilator-associated pneumonia (VAP), catheter-associated urinary tract infections (CAUTIs), and central line-associated bloodstream infections (CLABSIs) (6).

In Iran, the prevalence of NIs has been increasing in recent decades. Iran has a population of about 83 million people and a health system that comprises the public and private sectors. The public sector provides primary health care services through a network of health houses and health centers in rural areas and urban health posts in urban areas. The private sector mainly provides secondary and tertiary health care services through hospitals and clinics (7). The Ministry of Health and Medical Education (MOHME) is responsible for developing policies and guidelines for infection prevention and control (IPC) in healthcare facilities (8). Various studies have shown that the incidence of NIs in some healthcare facilities in the country is over 25% (9). In a study conducted in 2018 in various departments of public and private hospitals in Tehran, the prevalence of NIs was reported to be 26.4% (10). In another study conducted in 2019 in the surgical and ICU departments of hospitals in Tehran, the prevalence of NIs was 25.3% (11). In addition, a study conducted in 2016 in the ICU departments of hospitals in Tabriz estimated the prevalence of NIs to be 30.6% (12). The increasing trend of NIs in Iran underscores the need for a better understanding of antimicrobial resistance patterns and improving prevention and control strategies.

One of the main challenges in the field of NIs is the increasing prevalence of antimicrobial resistance in bacterial and fungal pathogens. In the past two decades, the rate of antibiotic resistance has increased significantly in bacteria such as *Escherichia coli*, *Staphylococcus aureus*, *Pseudomonas* *aeruginosa*, and Enterococcus (13-16). Some of the multidrug-resistant bacteria that cause NIs are methicillin-resistant *S. aureus* (*MRSA*), extended-spectrum beta-lactamase (ESBL)-producing *Enterobacteriaceae*, carbapenem-resistant *Acinetobacter baumannii*, and *vancomycin-resistant Enterococcus* (*VRE*) (17). These bacteria can acquire resistance genes from other bacteria through horizontal gene transfer or develop resistance mutations due to selective pressure from antibiotic use(18). Overuse or misuse of antibiotics, lack of infection control measures, and low awareness of healthcare workers and patients are some of the factors that contribute to the emergence and spread of antimicrobial resistance (19). This phenomenon leads to reduced efficacy of common antimicrobial drugs, resulting in longer treatment courses, increased costs, and even patient death (20). Therefore, continuous monitoring of antimicrobial resistance patterns in hospitals is essential. 

Imam Khomeini Hospital in Urmia, northwestern Iran, is a major healthcare facility that admits and treats a large number of patients each year. It is a 600-bed hospital that provides secondary and tertiary care services in various specialties, such as internal medicine, Surgery, orthopedics, eye, urology, otorhinolaryngology (ENT), neurosurgery internal nerve surgery, etc. It also serves as a teaching hospital for Urmia University of Medical Sciences. Given the importance of this healthcare facility and the need to understand antimicrobial resistance patterns for NI control, the present study was designed and conducted to investigate the multidrug resistance patterns of bacteria isolated from NIs in this facility. This study aimed to investigate multidrug-resistant bacterial strains in NIs at Imam Khomeini University Hospital in Urmia, Iran.

## Material and Methods

This was a cross-sectional study conducted using a convenience sampling method. A cross-sectional study was chosen because it allows measuring the prevalence and distribution of NIs and antimicrobial resistance patterns in a given population at a specific point in time. A convenience sampling method was chosen because it is easy and fast to perform in a busy hospital setting. However, these choices may limit the generalizability and causal inference of the findings. This study was approved by the Ethics Committee of Urmia University of Medical Sciences and informed consent was obtained from all patients or their legal guardians before sample collection (IR.UMSU.REC.1398.509).

The study population included all positive clinical samples from HAIs registered in the laboratory of Imam Khomeini Hospital, Urmia, Iran, in 2019. In this study, a total of 607 positive clinical samples from HAIs were evaluated. A HAI is defined as an infection that occurs during hospitalization or within 48 hours after discharge from the hospital. The inclusion criteria were: hospitalization of the patient for 48 to 72 hours, symptoms of infection at the time of admission or in the active phase of infection. The exclusion criteria included infection present or incubating at admission, and infection related to the previous hospitalization or surgery within 30 days before admission. 

Sampling was performed as follows: after the diagnosis of infection by the attending physician, the representative clinical samples were collected from the infected parts of the patient’s body using sterile swabs or containers. The type and volume of samples collected were blood (10 ml), urine (5 ml), sputum (2 ml), wound (1 ml), pleural fluid (5 ml), and other body fluids (5 ml). The samples were immediately sent to the laboratory in appropriate transport media under refrigerated conditions. The time interval between sample collection and processing was less than 4 hours.

**Bacterial Identification:**

In the process of bacterial identification, clinical samples were first cultured on differential media, specifically blood agar and MacConkey agar plates supplied by Oxoid. Blood agar, supplemented with 5% blood, was utilized to observe hemolytic reactions, while MacConkey agar facilitated the differentiation of lactose-fermenting bacteria from non-fermenters via color indicators.

After the incubation period, a series of standard biochemical assays were employed to ascertain the specific bacterial species present. The assays conducted were as follows:

Gram staining classifies bacteria based on the structural differences in their cell walls.

The catalase test distinguishes between bacteria that produce the enzyme catalase, such as Staphylococci and those that do not, like Streptococci.

The oxidase test, to identify bacteria containing cytochrome c oxidases.

The indole test, to detect the breakdown of tryptophan into indole by certain bacteria.

The citrate utilization test, to determine if bacteria can metabolize citrate as their sole carbon source.

The urease test, to detect the hydrolysis of urea by the urease enzyme.

These tests were interpreted in accordance with the guidelines outlined in Bergey’s Manual of Systematic Bacteriology. The incubation was carried out at a standard temperature of 37°C, ranging from 24 to 48 hours. The incubation atmosphere was tailored to the oxygen requirements of the isolated strains, with aerobic conditions applied generally, and anaerobic conditions for those bacteria requiring lower oxygen levels. Interpretation of the results was based on observable indicators such as pH-related color changes, gas production, and other metabolic byproducts, following the manufacturer’s protocols. This meticulous approach was critical for the precise identification of bacterial isolates.

**Antibiotic Susceptibility Testing**

Antibiotic susceptibility testing was performed using the disc diffusion method according to CLSI guidelines. The following antibiotics were tested using disc diffusion: ampicillin (10 mcg), amoxicillin-clavulanate (20/10 mcg), ceftriaxone (30 mcg), cefotaxime (30 mcg), ceftazidime (30 mcg), cefepime (30 mcg), imipenem (10 mcg), meropenem (10 mcg), gentamicin (10 mcg), amikacin (30 mcg), ciprofloxacin (5 mcg), levofloxacin (5 mcg), tetracycline (30 mcg), trimethoprim-sulfamethoxazole (1.25/23.75 mcg), nitrofurantoin (300 mcg), vancomycin (30 mcg), linezolid (30 mcg), and teicoplanin (30 mcg). The quality control procedures and the criteria for the interpretation of the results of the antibiotic sensitivity tests were as follows: the use of *E. coli* ATCC 25922 and *S. aureus* ATCC 25923 as reference strains, measuring the diameter of the area around each antibiotic disk, comparing the diameter of the area with the expected table. according to CLSI and classify bacteria as susceptible, moderate, or resistant. 

Data analysis was conducted using SPSS version 22 statistical software, employing descriptive statistical tests such as the mean, standard deviation, frequency, and percentage frequency. To compare the prevalence and resistance levels across different variable groups, statistical tests like the chi-square, t-test, ANOVA, and logistic regression were utilized. A P-value of less than 0.05 was deemed to indicate statistical significance. A 95% confidence interval was calculated for all estimates.

## Results

The data was collected from 607 clinical samples taken from different sites of the infection between January 2019 and December 2019.

The distribution of gender among the patient samples showed that 47.1% were male and 52.9% were female. Based on the distribution of age groups, 8.1% were in the age group of 1 to 25 years, 35.4% were in the age group of 25 to 50 years, 38.9% were in the age group of 50 to 75 years, and 17.6% were in the age group of 75 to 100 years. The mean age of the patients was 54 ± 20.4 years.

 The distribution of the samples by site of infection in the patients with hospital-acquired infection is shown in Table 1. The findings showed that the highest positive samples were in blood (269 samples), urine (137 samples), and sputum (91 samples), respectively. These three sites accounted for 85.69% of all samples. The lowest positive samples were in ascites (2 samples), tracheal tube culture (2 samples), and pericardial culture (2 samples), respectively. These three sites accounted for 1.37% of all samples.

Based on the frequency of distribution of the strains, E. coli was known to be the most common strain, accounting for 27.51% of the cases (Table 2).

In Table 3, resistance and sensitivity percentages of E. coli microorganisms isolated from the clinical samples have been categorized.

Our analysis reveals that *E. coli* samples exhibit a high prevalence of antibiotic resistance. The average resistance rate across all the antibiotics tested was 62.31%, whereas the average susceptibility rate was 47.53%. Standard deviations for both resistance (25.93) and susceptibility (26.46) highlighted a variation in these percentages among different antibiotics. 

Table 4 presents the resistance and sensitivity percentages of the Acinetobacter microorganisms isolated from the clinical samples.

**Table 1:** Frequency distribution of sample sites of the patients with hospital infection

location	Frequency	Percentage
Blood	269	46.38
Urine	137	23.62
Sputum	91	15.69
Wound	52	8.97
Pleural effusion	15	2.59
Other body fluids	8	1.38
Ascites	4	0.69
Tracheal tube culture	2	0.34
Pericardial culture	2	0.34
Total	580	100%

**Table 2:** Frequency distribution based on the strain type in samples of the patients with hospital infection

Percentage	Frequency	Strain type
**27.51**	167	***E. coli***
**18.45**	112	***Acinetobacter***
**15.15**	92	***Klebsiella***
**9.72**	59	***Pseudomonas***
**7.25**	44	***Citrobacter***
**5.11**	31	***S. aureus***
**4.45**	27	***S. epidermidis***
**2.47**	15	***Enterobacter***
**1.65**	10	***Enterococus***
**1.32**	8	***Staphylococcus saprophyticus***
**1.32**	8	***Streptococcus Group D***
**1.32**	8	***viridans streptococci***
**1.15**	7	***Providencia***
**1.15**	7	***Proteus spp.***
**0.66**	4	***Stenotrophomona***
**0.66**	4	***Flavobacterium***
**0.33**	2	***Serratia***
**0.33**	2	***Shigella sonnei***
**% 100**	607	**Total**

**Table 3:** Resistance and sensitivity percentage of *E. coli* microorganisms isolated from the clinical samples (%N)

Antibiotic	N	Resistance (%)	Sensitivity (%)
(Cotrimoxazole) SXT	155	93 (60)	62 (40)
(Ciprofloxacin) CP	116	84 (72.4)	32 (27.6)
(Cefotaxime) CTX	121	87 (71.9)	34 (28.1)
(Gentamicin) GM	153	62 (40.5)	91 (59.5)
(Piperacillin-tazobactam) PI.TZ	36	9 (25)	27 (75)
(Levofloxacin) LEVO	66	36 (54.5)	30 (45.5)
(Imipenem) IPM	146	61 (41.8)	85 (58.2)
(Ceftriaxone) CRO	51	36 (70.6)	15 (29.4)
(Piperacillin100mg) PIP	45	36 (80)	9 (20)
(Nitrofurantoin) FM	35	4 (11.4)	31 (88.6)
(Ceftazidime) CAZ	18	16 (88.9)	2 (11.1)
(Colistin) COL	2	100	2 (100)
(Ceftralaxone) CTR	2	100	-
(CEFEPIME) FEP	26	12 (46.2)	14 (53.8)
Ampicillin sulbactam	14	10 (71.4)	4 (28.6)

**Table 4:** Percentage of resistance and sensitivity of the *Acinetobacter* microorganisms isolated from the clinical samples, (N)%

Antibiotic	N	Resistance (%)	Sensitivity (%)
Co-trimoxazole (SXT)	97	95.9 (93)	4.1 (4)
Ciprofloxacin (CP)	87	100 (87)	0 (0)
Cefotaxime (CTX)	89	100 (89)	0 (0)
Gentamicin (GM)	105	86.7 (91)	13.3 (14)
Piperacillin-tazobactam (PI.TZ)	9	100 (9)	0 (0)
Levofloxacin (LEVO)	66	90.9 (60)	6.1 (4)
Imipenem (IPM)	95	41.8 (61)	58.2 (84)
Ceftriaxone (CRO)	22	100(22)	0
Cefoxitin (CFO)	2	100(2)	0
Linezolid (LINE)	2	100(2)	0
Penicillin (P)	2	100(2)	0
Erythromycin (E)	2	100(2)	0
Clindamycin (CC)	4	100(4)	0
Piperacillin (PIP)	28	7.1(2)	92.9(26)
Nitrofurantoin (FM)	10	20(2)	80(8)
Ceftazidime (CAZ)	4	100(4)	0
Meropenem (MERO)	2	100(2)	0
Colistin (COL)	6	100(6)	0
Cefepime (FEP)	2	100(2)	0
Ampicillin-sulbactam)	2	100(2)	0

Analysis of antibiotic resistance and sensitivity data from the clinical Acinetobacter isolates reveals an alarming trend. The mean resistance rate across all antibiotics tested was a staggering 87.12%, with a median resistance of 100%. This signifies that a significant portion of these bacteria exhibit complete resistance (100%) to the antibiotics used in this study. Conversely, the mean sensitivity was a mere 12.73%, with a median of 0%. These findings highlighted the concerning prevalence of multidrug-resistant Acinetobacter, posing a significant challenge in treating infections caused by these bacteria.

 The frequency and antibiotic resistance percentage of the bacteria in the hospital for each drug are listed in Table 5:

**Table 5:** Investigating the distribution of the antibiotic resistance frequencies in the samples submitted from the patients with hospital infections

Antibiotic	Frequency	Antibiotic Resistance (%)	Antibiotic	Frequency	Antibiotic Resistance (%)
Ciprofloxacin (CP)	331	77.9%	Ceftriaxone (CRO)	155	88.1
Levofloxacin (LEVO)	144	69.2%	Gentamicin (GM)	328	60.5
Cotrimoxazole (SXT)	410	80.1%	Cefotaxime (CTX)	358	86.5
Imipenem (IPM)	303	68.7%	Piperacillin	99	81.1
Nitrofurantoin (FM)	24	33.8%	Cefepime (FEP)	70	72.9
Piperacillin-tazobactam (PI.TZ)	74	59.2%	Tetracycline (TE)	28	56
Erythromycin (E)	50	80.6%	Amoxicillin (AMX)	3	100
Rifampin (RIF)	14	73.7%	Doxycycline (DOX)	9	69.2
Linezolid (LINE)	0	0%	Cefoxitin (CFO)	28	62.2
Penicillin (P)	66	97.1%	Meropenem (MERO)	12	75
Clindamycin (CC)	33	71.7%	Ofloxacin (OFX)	2	100
Ceftazidime (CAZ)	28	87.5%	Vancomycin (V)	14	77.8
Cefixime (CFM)	0	0%	Ceftriaxone (CTR)	2	100
Cephalexin (CF)	3	100%	Furazolidone (FURAZ)	2	100
Colistin (COL)	18	75%	Ampicillin-Sulbactam	24	80/00%

The distribution of antibiotic resistance in bacterial isolates from patients with HAIs was investigated. The results showed that 19.8% of the isolates were resistant to one antibiotic, 13.2% were resistant to three antibiotics, and 40.5% were not resistant to any antibiotics (Table 6).

 Analysis of the data showed that 91.3% of the isolates were resistant to four antibiotics or fewer, and 8.7% were resistant to more than four antibiotics.

**Table 6:** Distribution of resistance to the number of antibiotics in the bacterial strains isolated from submitted samples

Resistance to the number of antibiotics	Frequency	Percentage
0	246	40.5%
1	120	19.8%
2	65	10.7%
3	80	13.2%
4	43	7.1%
5	18	3%
6	33	5.4%
7	2	0.3%
Total	607	100%

## Discussion

NIs pose a significant threat to patient safety and public health, particularly in the era of antimicrobial resistance. The emergence of multidrug-resistant bacteria has further complicated the management of NIs, leading to increased morbidity, mortality, and healthcare costs. In this context, understanding the epidemiology of multidrug-resistant bacteria in hospital settings is crucial for developing effective infection control strategies and improving patient outcomes.

According to the findings of this study, the most common nosocomial infection was reported from the blood site. This finding is consistent with some other studies. For example, a study conducted in Tehran University Hospitals showed that blood infections with 20.71% were the most common type of nosocomial infection (6). In addition, a study conducted in Bojnurd showed that blood infection with 28.14% was the second most common nosocomial infection (21). However, in some other studies, blood infection has ranked lower. For example, a study performed in Isfahan University Hospitals showed that blood infection with 15.6% was the second most common nosocomial infection (22) and a study conducted in Tehran reported that as the fourth most common hospital infection (23). However, when compared to global data, the prevalence of NIs may vary. A systematic review and meta-analysis published in PLOS ONE found that the global prevalence of NIs is 0.14 %, with an annual increase of 0.06 % (24). The highest rates were observed in the African region, while the lowest were in the Americas and Western Pacific regions. The study also noted that *E. coli* was the most common microorganism in these infections, which differs from our findings where *E. coli*, *Acinetobacter baumannii*, and *Klebsiella pneumoniae* were the most isolated microorganisms. Additionally, the WHO has reported that the burden of healthcare-associated infection is several-fold higher in low- and middle-income countries than in high-income ones (25). These discrepancies highlight the influence of regional factors such as economic status, healthcare facilities’ conditions, and infection control practices. Therefore, there is a need for comprehensive and integrated studies that use standard and comparable methods to investigate the prevalence and causes of NIs at the national and regional levels.

The findings of this study emphasize *E. coli* as the predominant cause of NIs in Urmia, Iran, consistent with local studies conducted at Ilam and Isfahan Universities of Medical Sciences. Collectively, these studies underscore the significant presence of *E. coli* in clinical samples and urinary tract infections within the region (26), (27). 

A broader examination of the global landscape reveals a similar pattern; a study published in 2023 identified *E. coli* as the most frequent pathogen in NIs, followed by microorganisms such as Coagulase-negative staphylococci. Furthermore, a study published in 2024 on Haemophilus influenzae reported a significant prevalence of multi-drug resistant strains, particularly in Asian countries (28). A Polish study highlighted the incidence of multi-drug resistant bacteria in a tertiary hospital, with a notable resistance mechanism being extended-spectrum beta-lactamase (ESBL) production by *Klebsiella spp*., *E. coli*, or *Enterobacter spp*. Isolates (29). Additionally, the rising challenge of superbugs, which exhibit resistance to multiple antibiotics, poses a severe threat to patient safety worldwide (30).

These international findings corroborate the high prevalence of *E. coli* observed in our study and emphasize the global challenge posed by multi-drug resistant bacteria in healthcare settings. Therefore, healthcare facilities worldwide must implement more rigorous preventive and infection control measures to prevent the transmission and spread of these pathogens, ultimately ensuring patient safety and public health.

Based on the findings of this study, the percentage of resistance in *Escherichia coli* pathogens was relatively high, especially against SXT, CP, CTX, and CRO antibiotics, which showed the highest percentage of resistance. Some other studies have also shown a parallel increase in the percentage of resistance of *E. coli* to various types of antibiotics, including dysbiotics and chemicals (31-33). Given these results, the use of specific antibiotics for the treatment of *Escherichia coli* pathogens is facing significant challenges, and this shows that the use of new and advanced methods in the treatment of *E. coli* pathogens is essential.

The results of this study showed that resistance to various antibiotics in the samples was very high. In particular, resistance to CP, AMX, SXT, PI, and E was more than 80%. These results show that hospitals, as an environment with the highest prevalence of infections caused by antibiotic-resistant bacteria, can play an important role in demonstrating the problems of creating antibiotic resistance in the community and other health sectors. Some recent studies have also shown that antibiotic resistance is one of the major therapeutic challenges that occurs in hospitals, especially in patients who are hospitalized for a long time (34-38).

In this study, antibiotic resistance in bacterial samples was relatively high, and a significant number of samples were resistant to at least two or three antibiotics. A study conducted in various hospitals around the world showed that the rate of antibiotic resistance in multiple bacteria, including *E. coli*, *Pneumococcus*, *Staphylococcus*, and *Enterococci*, has increased in recent years, which is consistent with the results of our study (39). These findings show that multidrug-resistant bacterial infections are a new and serious challenge in medicine and public health and require comprehensive and coordinated strategies for their control and prevention. The scientific and practical value of this study is in identifying the pattern of antibiotic resistance in the bacteria that cause NIs, which can help in determining appropriate and effective treatment regimens for the patients.

## Conclusion

The results of this study showed that NIs are a serious public health issue at Imam Khomeini University Hospital in Urmia. These infections require urgent and comprehensive action to reduce their prevalence and severity. The following recommendations are proposed to address this issue:

- Implementation of sanitary standards and technical requirements in all patient and treatment units of the hospital.
- Rapid and accurate diagnosis of the cause of infection before starting antibiotic therapy and selection of the appropriate antibiotic based on bacterial sensitivity.
- Reduction of unnecessary and inappropriate use of antibiotics and compliance with their use instructions.
- Education and awareness of physicians, nurses, pharmacists, and patients about the risks of NIs and methods to prevent them.
- Implementation of a nosocomial infection surveillance system and periodic reporting of key infection indicators.

## Limitations

The limitations of this study include its cross-sectional nature and the fact that it was conducted in only one hospital.
